# Genetic analysis of loose smut (*Ustilago tritici*) resistance in Sonop spring wheat

**DOI:** 10.1186/s12870-020-02525-x

**Published:** 2020-07-03

**Authors:** Dinushika Thambugala, Jim G. Menzies, Ron E. Knox, Heather L. Campbell, Curt A. McCartney

**Affiliations:** 1 Agriculture and Agri-Food Canada, Morden Research and Development Centre, Morden, MB Canada; 2 Agriculture and Agri-Food Canada, Swift Current Research and Development Centre, Swift Current, SK Canada

**Keywords:** Disease resistance, Sonop (TD-14), *Triticum aestivum*, Loose smut, *Ustilago tritici*, Linkage analysis, Quantitative trait locus (QTL)

## Abstract

**Background:**

The genetics of resistance to loose smut of wheat (*Triticum aestivum L*.) caused by the fungus *Ustilago tritici* (Pers.) Rostr. is not well understood. This study examines loose smut resistance in Sonop (TD-14), a South African spring wheat variety. A doubled haploid (DH) population of 163 lines derived from the cross Diamont/TD-14 was studied. The parents and progenies were inoculated with *U. tritici* races T2, T9, and T39 individually in growth facilities at Morden and Swift Current, Canada. Loose smut incidence (LSI) and partial loose smut resistance (PLSR) were assessed.

**Results:**

A whole genome linkage map was developed consisting of 11,519 SNP loci found on 31 linkage groups spanning 2845 cM. A new major resistance gene *Ut11* was located to the distal end of chromosome arm 7BS. *Ut11* conferred resistance to *U. tritici* race T2, but not races T9 and T39. Quantitative trait locus (QTL) mapping identified four QTL controlling LSI in the Diamont/TD-14 DH population on chromosomes 3B, 4B, 5B, and 7B (at *Ut11*) with TD-14 contributing the resistance alleles at three of these loci. The major QTL *QUt.mrc-5B* was effective against all three races and explained up to 81% of the phenotypic variation. The only QTL identified for PLSR coincided with the LSI QTL *QUt.mrc-5B* indicating that this locus affected both loose smut incidence and partial smutting of spikes.

**Conclusions:**

A race-specific resistance gene *Ut11* and a broadly effective resistance QTL *QUt.mrc-5B* were the main loci controlling loose smut resistance in the differential line TD-14 (cultivar Sonop). This study provides insight into the genetics of loose smut resistance in spring wheat Sonop and the single nucleotide polymorphism (SNP) markers linked to the resistance gene *Ut11* and QTL *QUt.mrc-5B* will be useful for selecting loose smut resistance in breeding programs.

## Background

Loose smut is a common disease throughout the wheat-growing regions of the world that is caused by the basidiomycete fungus *Ustilago tritici* (Pers.) Rostr [1]. This seed-borne disease is commonly present in wheat fields of western Canada at low levels of incidence [2, 3] and is also common in the United States [2]. Although loose smut is currently managed in western Canada with a combination of resistant cultivars, certified seed, and systemic fungicides applied as seed treatments [4], it can cause significant yield and economic losses in the absence of effective control practices [5]. The development of resistant cultivars is the most desirable and environmentally friendly strategy of managing this disease over other means of control [6, 7]. Further, the development and production of loose smut-resistant wheat cultivars is particularly important in organic wheat production and in countries where seed treatment is not readily available [7].

*U. tritici* races of differing virulence have been reported from both hexaploid and durum wheat worldwide. Approximately fifty races of *U. tritici* have been identified from various regions of the world growing hexaploid wheat [7–10]. The virulence of the Canadian population of *U. tritici* varies considerably [11]. For instance, races such as T9, T10, and T39 are virulent on many hexaploid wheat lines in the Canadian differential host series, whereas races such as T5, T6 and T56 possess virulence on one or few wheat lines [9]. Because new races of *U. tritici* continue to be identified in commercial wheat fields in Canada, it is important to identify new resistance genes and understand the mechanisms of loose smut resistance in wheat [11, 12].

Previous studies on the genetics or mechanisms of loose smut resistance in wheat have shown that resistance may be inherited as a qualitative or quantitative trait [13, 14]. However, the majority of genetic studies carried out thus far have demonstrated simple inheritance of loose smut resistance with one, two or three major genes in hexaploid wheat controlling resistance to several races of *U. tritici* [15–18]. The first four loose smut resistance genes *Ut1* to *4* were named based on segregation of avirulence in *U. tritici* [19, 20]. Genes *Ut1* and *Ut3* have no chromosome assignment. Based on pedigree, the gene symbol *Ut2* was assigned to the resistance gene on chromosome 6A to race T19 [18]. *Ut4* associated with the Thatcher derived differential line TD12A, was located on chromosome 7B [14, 21]. *Ut5* initially identified as *Ut-X* was located on chromosome 2BL [22]. *Ut6* was initially located on chromosome 5B by Kassa et al. [23] and confirmed by Knox et al. [14]. A gene located to chromosome 7A by Dhitaphichit et al. [24] was subsequently named *Ut7* [14]. Knox et al. [14] further identified genes *Ut8* on chromosome 3A, *Ut9* on chromosome 6B and *Ut10* on chromosome 6D. Several studies revealed the additive nature of resistance genes, while in some cases, duplicate complementary action of multiple genes was also implicated [25].

Although loose smut resistant wheat varieties have been developed and grown, very few studies have focussed on identification and mapping of genomic regions controlling resistance to *U. tritici*. Opportunities for the utilization of alternative sources of loose smut resistance, such as that in Sonop, exist by the identification of QTL or genes underlying the resistance. The incorporation of previously unidentified resistance into well-adapted wheat cultivars diversifies resistance and is desirable over chemical control measures. The present study aimed to enhance the knowledge of the loose smut resistance in Sonop (TD-14), a South African spring wheat variety that carries a high level of resistance to loose smut and is a component of the Canadian loose smut differential set [8]. The resistance phenotype of Sonop ranges from partially smutted spikes to completely healthy spikes. The present study characterized the complete and partial loose smut resistance in a DH population of 163 individuals developed from the cross Diamont/TD-14. QTL mapping was used to determine the chromosomal location of resistance gene(s). Through linkage and QTL mapping, molecular markers that are suitable for marker-assisted selection were identified.

## Results

### Phenotypic analysis of loose smut resistance

The resistant parent TD-14 showed nearly complete resistance to *U. tritici* races T2, T9 and T39, whereas the susceptible parent Diamont was highly susceptible to all three races (Fig. 2; Table 1). The frequency distributions of loose smut incidence (%) to races T9 and T39 were continuous and bimodal, whereas the Diamont/TD-14 DH population was strongly skewed with a high frequency of lines with resistance when tested with race T2 (Fig. 2a). Based on the race T2 data, the DH lines were classified into resistant and susceptible classes as outlined by Nielsen [8], which considered wheat lines with 0–10% LSI as resistant and wheat lines with > 10% LSI as susceptible [11, 23]. Based on these criteria, there were 81 lines resistant and 87 susceptible to race T2. The ratio of resistant to susceptible DH lines with race T2 was consistent with single-gene segregation (χ^2^ = 0.15, *P* = 0.70) in the Diamont/TD-14 DH population.

**Table 1:** Descriptive statistics of the Diamont/TD-14 DH population for loose smut incidence (LSI, %) and fully smutted index (FSI, %)

Trait	Population			Parents	
	Mean	Min^**a**^	Max^**b**^	Diamont	TD-14
**LSI (%)**					
T2_Morden	27	0	100	56	0
T2_Swift	41	0	100	92	0
T2_Pooled	34	0	99	74	0
T9_Morden	46	0	100	64	20
T9_Swift	52	0	100	96	7
T9_Pooled	49	0	100	80	14
T39_Morden	35	0	96	59	3
T39_Swift	46	0	100	92	1
T39_Pooled	41	0	96	76	2
**FSI (%)**					
T2_Morden	60	0	100	100	nd^c^
T2_Swift	52	0	100	100	nd
T2_Pooled	54	0	100	100	nd
T9_Morden	65	0	100	100	0
T9_Swift	65	0	100	100	nd
T9_Pooled	63	0	100	100	nd
T39_Morden	81	0	100	100	nd
T39_Swift	75	0	100	100	nd
T39_Pooled	76	0	100	100	nd

^a^*Min* minimum, ^b^*Max* maximum, ^c^*nd* not determined since LSI was < 20%

**Fig. 1:**
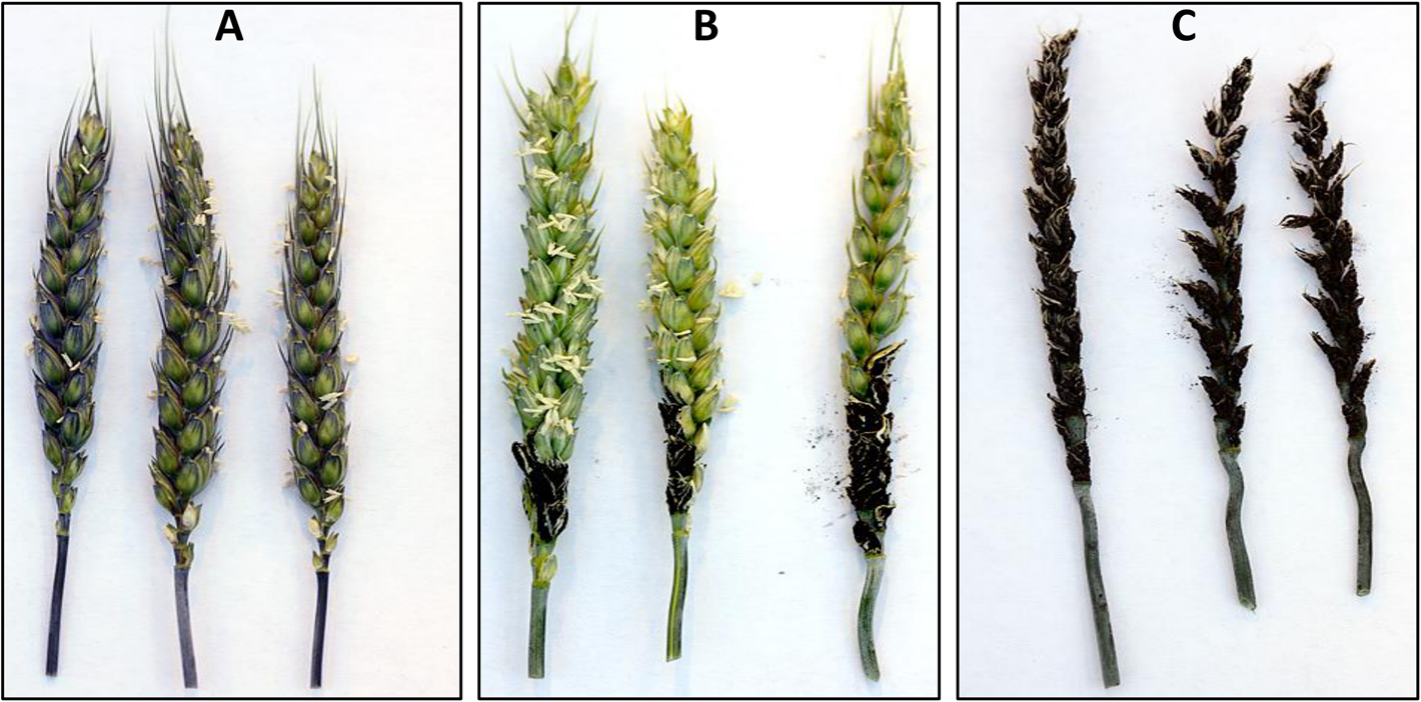
Loose smut symptoms caused by *Ustilago tritici* on common spring wheat: **a** resistant, **b** partially resistant, and **c** susceptible (fully smutted)

**Fig. 2:**
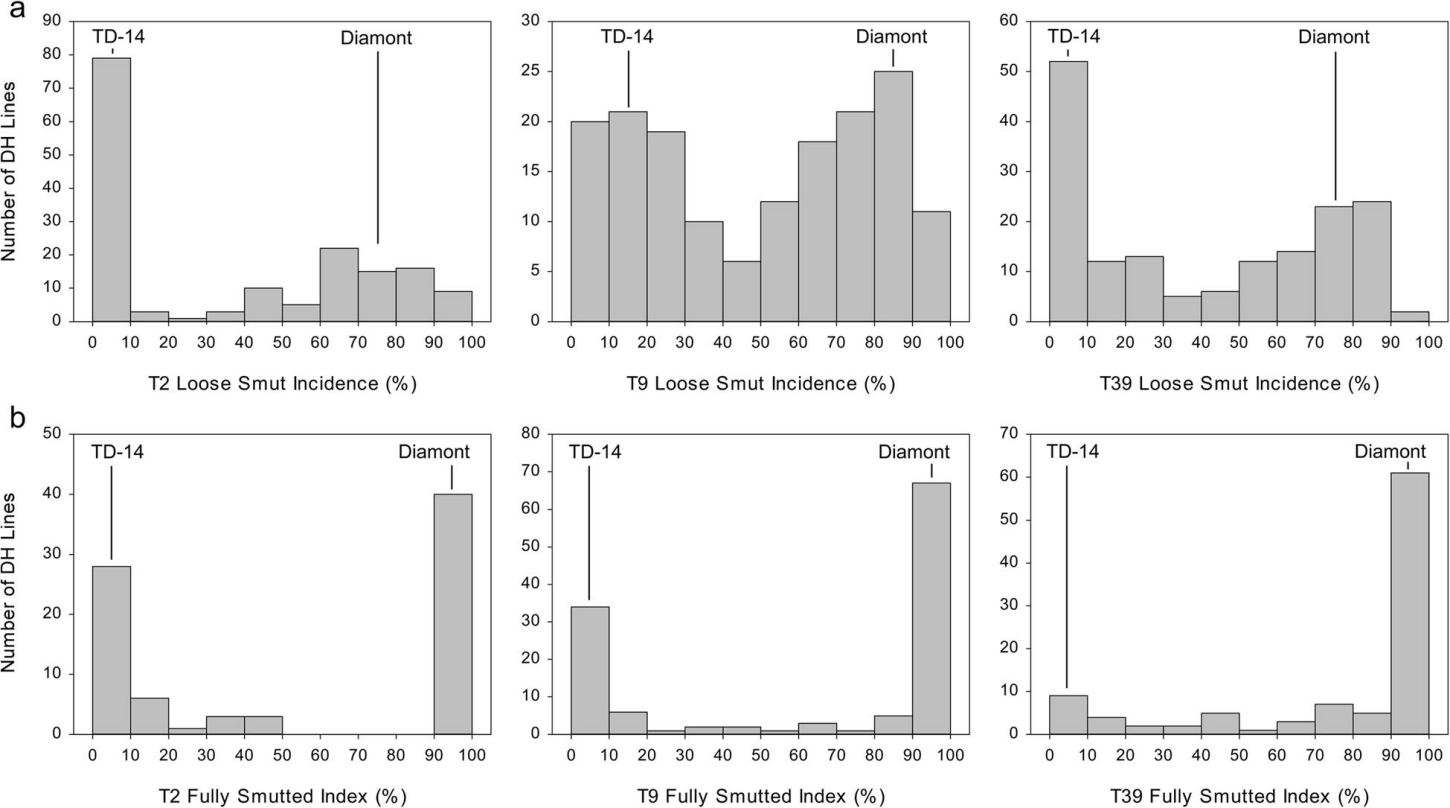
Frequency distribution of **a** loose smut incidence (LSI %) and **b** fully smutted index (FSI %) within the Diamont/TD-14 DH mapping population in response to *Ustilago tritici* individual races T2, T9 and T39 based upon the pooled dataset. Values for parents Diamont and TD-14 are indicated

When the reactions of DH lines to races T2, T9 and T39 were considered together, two resistant phenotypes were observed in the Diamont/TD-14 DH population: (1) resistant with healthy heads and (2) partially resistant with partially smutted heads (Fig. 1). The distributions of FSI among DH lines were bi-modal with a few intermediates between resistant and susceptible DHs for *U. tritici* races T2 and T9 (Fig. 2b).

### High-density genetic map

A high-density whole genome linkage map was developed for the Diamont/TD-14 DH population using the 90 K wheat Infinium SNP chip. The complete linkage map is reported in Table S1. The Diamont/TD-14 linkage map consisted of 11,519 loci. The total map length across the 31 linkage groups was 2845 cM. Some chromosomes consisted of more than one linkage group, which were consecutively numbered starting at the distal end of the short arm. The percentage of loci mapping to the A, B, and D genomes were 38.4, 54.3, and 7.3%, respectively (Table S2). Group 4 chromosomes consisted of considerably fewer loci (7.0%) than the other chromosome groups (average of 15.5%). Overall, there were 4.0 loci/cM.

The qualitative data for race T2 enabled the mapping of a new loose smut resistance gene, designated as *Ut11*, to the distal end of chromosome arm 7BS (Fig. 4, Table S1). Comparative mapping revealed that *Ut11* and *Ut4* were distinct. *Ut4* is the only other described loose smut resistance gene on chromosome 7B, and was located on the long arm [14, 21]. *Ut11* co-segregated with SNP markers BS00022562_51, Excalibur_c3489_182, and Kukri_rep_c71778_644, which have BLAST locations of 0.43, 1.20, and 1.25 Mbp, respectively, on chromosome 7B in IWGSC Chinese Spring RefSeq v1.0 [26]. The *Ut4* linked markers *gwm302* and *wmc723* [14] have BLAST locations of 633.12 Mbp. This projects the *Ut4* locus to approximately 82 cM on the 7B linkage map of the Diamont/TD-14 DH population based upon the BLAST locations of the SNP loci Ku_c5351_1820 (622.27 Mbp), Excalibur_c17686_554 (639.36 Mbp), and Excalibur_c33549_95 (642.28 Mbp). The 7B linkage map in the Diamont/TD-14 DH population was 151.5 cM in length and spanned the entire length of the chromosome based upon BLAST locations of the SNP loci in the reference genome sequence.

A reference genetic stock (DH line TD14XDIA*B0075) for the newly identified loose smut resistance gene *Ut11* is archived at the Plant Gene Resources of Canada (PGRC) genebank as accession CN 120264. This DH line carries the resistance gene *Ut11*, which confers resistance to *Ustilago tritici* T2, but is susceptible to races T9 and T39. Based on the available data, *Ut11* is the only loose smut resistance gene present in TD14XDIA*B0075.

### QTL for loose smut incidence

QTL detected based on LSI contained ‘mrc’ in their name whereas QTL identified for PLSR contained ‘p.mrc’. A total of four additive effect QTL for loose smut resistance were identified in the Diamont/TD-14 DH population based upon IM and ICIM [27, 28]. These QTL were detected on chromosomes 3B, 4B, 5B, and 7B, and were designated *QUt.mrc-3B*, *QUt.mrc-4B*, *QUt.mrc-5B*, and *QUt.mrc-7B*, respectively. Table 2 presents the chromosomal positions of QTL, LOD scores of QTL peaks, the proportion of phenotypic variation explained (% PVE) and favourable parental allele. TD-14 alleles contributed to the resistance at three of these QTL, which is consistent with TD-14 being resistant to loose smut. Diamont contributed a single minor QTL on chromosome 4B (*QUt.mrc-4B*).

**Table 2:** Additive effect QTL identified for resistance to loose smut (*Ustilago tritici*) in the Diamont/TD-14 DH population

QTL designation	Race - location combinations	Chr^**c**^	IM^**a**^				ICIM^**b**^			
			Pos^**d**^	LOD^**e,f**^	PVE^**g**^	Add^**h**^	Pos	LOD	PVE	Add
**Loose Smut Incidence (LSI)**										
***QUt.mrc-3B***	T9_Swift	3B.1	78.0	4.21	5.64	12.19	76.2	3.92	2.61	5.82
	T9_Mean	3B.1	78.0	3.86	5.31	9.98				
	T39_Swift	3B.1	76.8	3.03	4.21	11.31				
	T39_Mean	3B.1	76.8	3.56	4.80	10.11				
***QUt.mrc-4B***	T9_Swift	4B					69.3	3.26	2.14	−5.32
	T9_Mean	4B					69.9	3.81	3.09	−5.51
	T39_Mean	4B					61.3	2.51	1.48	−4.08
***QUt.mrc-5B***	T2_Morden	5B.1					19.8	5.74	3.78	6.07
	T2_Swift	5B.1					22.2	8.39	2.26	6.21
	T2_Mean	5B.1					19.8	7.89	2.74	5.92
	T9_Morden	5B.1	20.1	26.31	36.72	20.77	19.8	26.52	52.48	20.07
	T9_Swift	5B.1	20.4	41.89	35.16	30.39	20.4	50.53	68.61	29.74
	T9_Mean	5B.1	20.4	40.99	35.52	25.79	20.3	44.49	67.78	25.59
	T39_Morden	5B.1	19.8	26.75	23.78	21.09	19.8	27.61	54.16	20.29
	T39_Swift	5B.1	20.4	65.25	43.31	36.21	20.4	44.28	81.31	23.04
	T39_Mean	5B.1	20.4	52.12	38.74	28.65	20.4	56.05	76.44	28.79
***QUt.mrc-7B***	T2_Morden	7B	0.0	49.58	75.42	27.39	0.0	53.46	75.69	27.13
	T2_Swift	7B	0.0	78.71	89.24	39.33	0.0	86.49	88.89	38.93
	T2_Mean	7B	0.0	70.85	86.49	33.45	0.0	77.28	86.47	33.19
**Fully Smutted Index (FSI)**										
***QUtp.mrc-5B***	T2_Swift	5B.1	20.4	37.83	64.25	44.39	20.4	37.83	85.22	44.39
	T2_Pooled	5B.1	20.4	32.19	55.70	42.61	20.4	32.19	81.69	42.61
	T9_Morden	5B.1	21.0	55.94	78.29	42.87	21.0	58.48	88.16	43.16
	T9_Swift	5B.1	21.0	59.31	63.95	45.59	21.0	59.31	91.29	45.59
	T9_Pooled	5B.1	21.0	74.96	94.11	44.40	21.0	77.31	94.10	44.35
	T39_Morden	5B.1	21.0	24.78	69.18	32.34	21.0	24.78	69.18	32.34
	T39_Swift	5B.1	21.0	36.11	64.50	43.55	21.0	54.90	38.05	43.41
	T39_Pooled	5B.1	21.0	36.80	82.00	36.32	21.0	36.80	82.00	36.32

^a^*IM* interval mapping, ^b^*ICIM* inclusive composite interval mapping, ^c^*Chr* chromosome, ^d^*Pos* position in cM, ^e^*LOD* peak LOD score, ^f^*LOD* threshold for declaring significant QTL was 3.2, ^g^*PVE* phenotypic variation explained (r^2^, %), ^h^*Add* additive effect of allele substitution. Positive number indicates that TD-14 contributed the resistance allele

**Fig. 3:**
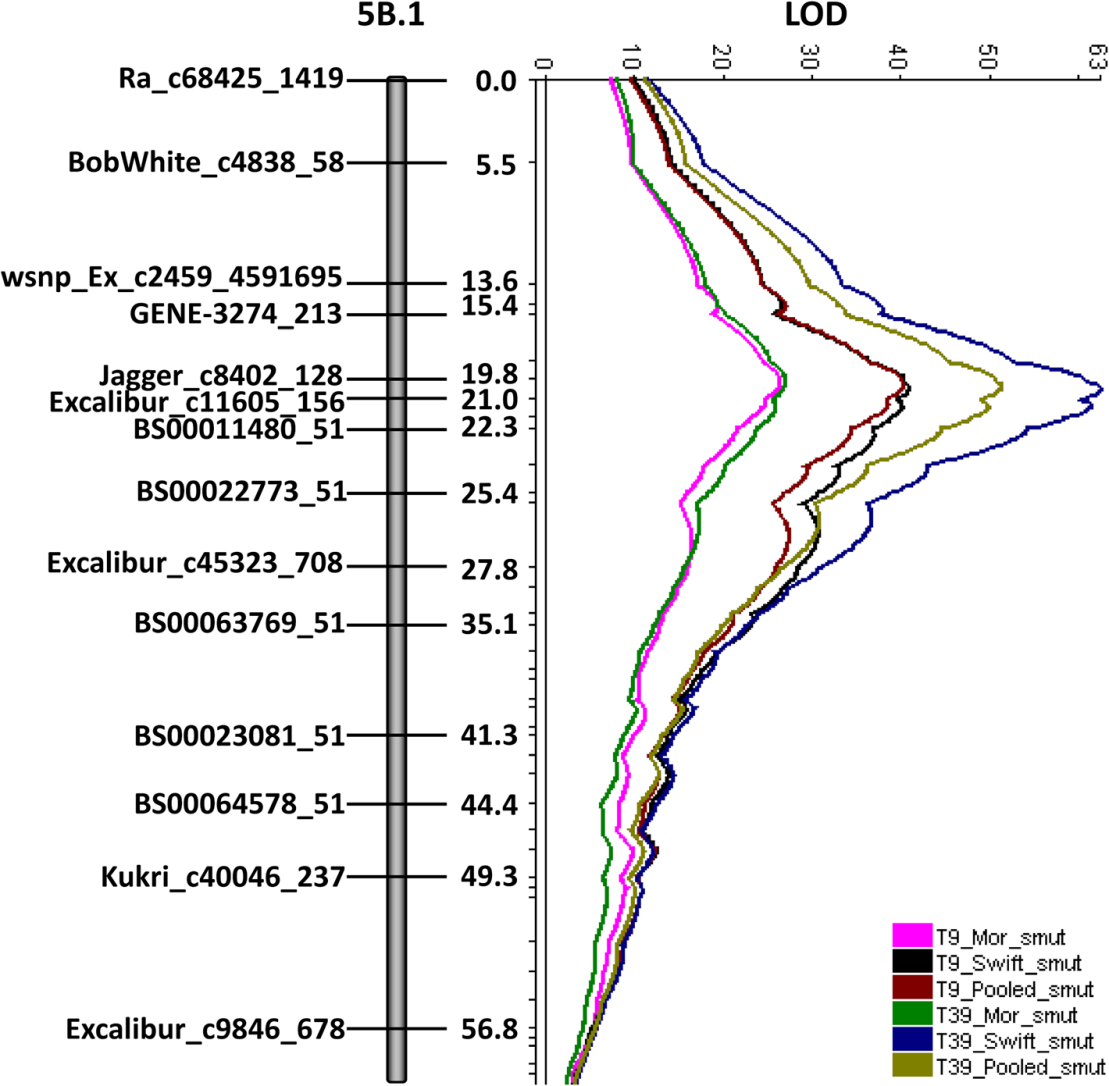
QTL *QUt.mrc-5B* region on chromosome 5B (linkage group 5B.1) associated with resistance to *U. tritici* races T9 and T39. Displayed LOD scans are based upon interval mapping. Map distances are in centiMorgans (cM). The LOD threshold value for declaring QTL was 3.2. Mor = Morden, Swift = Swift Current

A major and stable QTL, *QUt.mrc-5B*, was detected on chromosome 5B at 20.4 cM (linkage group 5B.1) for resistance to all three *U. tritici* races (Fig. 3). The BLAST locations of the SNP loci linked to *QUt.mrc-5B* correspond to 15.08 to 19.44 Mbp on chromosome 5B of IWGSC Chinese Spring RefSeq v1.0. *Ut6*-linked markers *barc142*, *barc266*, and *barc232* [14, 23] correspond to 605.52, 618.54, and 619.84 Mbp on chromosome 5B of IWGSC Chinese Spring RefSeq v1.0 [26], and corresponded to SNP RAC875_c28645_455 (616.65 Mbp) at 123 cM on the 5B.1 linkage map of the Diamont/TD-14 DH population. Therefore, *QUt.mrc-5B* and *Ut6* were genetically unlinked since they are approximately 100 cM apart on Diamont/TD-14 linkage group 5B.1. The parent TD-14 contributed resistance at *QUt.mrc-5B* and this QTL allele was consistent in accounting for a significant reduction in loose smut incidence for all the three races over the two experimental repetitions (Table 3). The maximum phenotypic variance explained (PVE) was 81.3% for *U. tritici* race T39, 68.6% for race T9, and 3.8% for race T2 (Table 2).

**Table 3:** Loose smut incidence (LSI, %) associated with resistance QTL *QUt.mrc-3B* (BS00030581_51), *QUt.mrc-4B* (JD_c11606_1380), *QUt.mrc-5B* (Excalibur_c11605_156), and *QUt.mrc-7B* (Kukri_rep_c71778_644) within the Diamont/TD-14 DH population for individual *Ustilago tritici* races T2, T9 and T39

QTL	Allele	Mean loose smut incidence (%)		
		T2	T9	T39
*QUt.mrc-3B*	Diamont	39	58	50
	TD-14	30	39	31
*QUt.mrc-4B*	Diamont	35	47	40
	TD-14	34	52	42
*QUt.mrc-5B*	Diamont	43	75	69
	TD-14	27	24	13
*QUt.mrc-7B*	Diamont	68	49	41
	TD-14	1	49	40

**Fig. 4:**
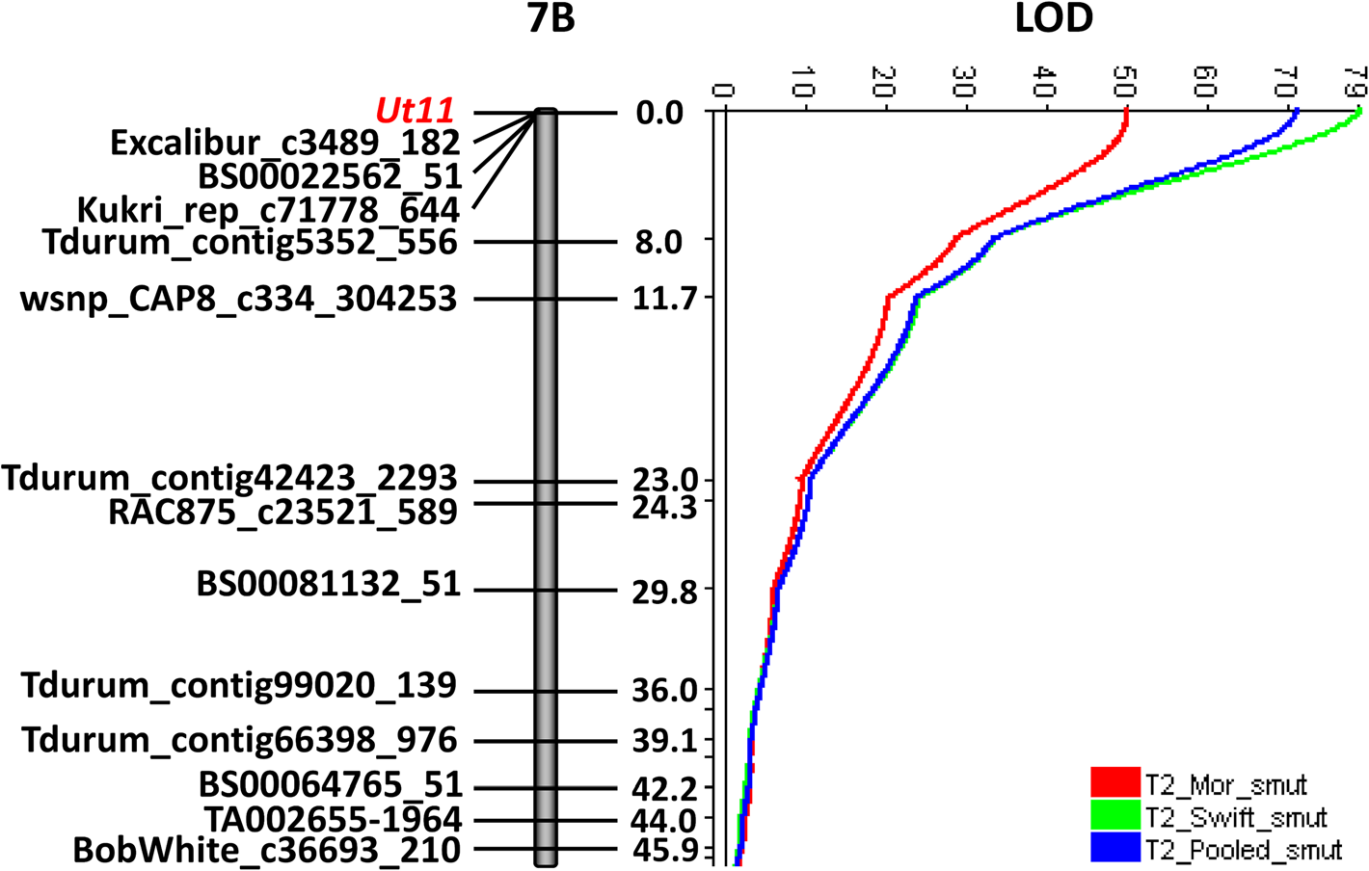
Location of the loose smut resistance gene *Ut11* on the distal end of chromosome arm 7BS. The QTL LOD scan is presented on the right showing specificity for *U. tritici* race T2. The LOD threshold value for declaring QTL was 3.2. Map distances are shown in centiMorgans (cM). Mor = Morden, Swift = Swift Current

Another significant QTL, designated as *QUt.mrc-7B*, mapped to the location of *Ut11* on chromosome 7B (Fig. 4). This QTL was detected only by race T2, which was the *U. tritici* race used to map *Ut11*. This QTL explained up to 89% of the phenotypic variation and maximum reduction in loose smut incidence when inoculated with race T2 (Tables 2 and 3). These QTL analysis results support the decision to map resistance to race T2 as a Mendelian factor. The minor QTL *QUt.mrc-3B* on the chromosome 3B (linkage group 3B.1) was identified for resistance to both races T9 and T39 (Table 2). Finally, the minor QTL *QUt.mrc-4B* on chromosome 4B (66.9 cM) was effective against races T9 and T39, with the resistant allele contributed by the susceptible parent Diamont (Table 2).

The only QTL identified for PLSR (*QUtp.mrc-5B*) based on FSI coincided with the major effect QTL *QUt.mrc-5B* identified based on LSI (Table 2). This PLSR QTL was effective against all three *U. tritici* races in the inoculations conducted in Morden and Swift Current. The LOD and PVE statistics were very high (Table 2) and reflected the bi-modal FSI distributions observed for each race (Fig. 2b). Race T2 had higher PVE statistics with FSI compared with LSI (Table 2).

Stable epistatic interactions were also detected for all three *U. tritici* races: T2, T9 and T39 (Table S3). Nine digenic epistatic interactions were identified between loci on chromosomes 1B and 7A, 2B and 6B, 2D and 6B, 2D and 7D.2, 3A and 6B, 3B.1 and 7A, 4D.1 and 6B, 4D.2 and 5B.1, and 5B.1 and 7B with the QIME module (i.e. interval mapping) (Table S3). However, only the 5B.1 and 7B epistatic interaction was significant with the QICE module (i.e. inclusive composite interval mapping). The 5B.1 and 7B loci were also reported as major additive effect QTL for loose smut resistance on chromosomes 5B (*QUt.mrc-5B*) and 7B (*Ut11*; *QUt.mrc-7B*) (Table 2). Epistatic interaction involving 5B.1 and 7B was identified only with race T2, which was the only race avirulent to *Ut11*.

## Discussion

The complete resistance phenotype of TD-14 to *U. tritici* race T2 and near complete resistance to races T9 and T39 indicated that TD-14 possesses a very effective form of resistance to all three races. However, only one of the three TD-14-derived resistance QTL was effective against all three *U. tritici* races. The *QUt.mrc-5B* on chromosome 5B was a major-effect QTL, because it contributed to loose smut resistance against all three *U. tritici* races and explained up to 81% of the phenotypic variation. This region of chromosome 5B was located on the short arm in contrast to *Ut6* the source of the resistance QTL *QUt.spa-5B* in cultivars Glenlea and AC Vista located on chromosome arm 5BL [14, 23]. The location of the TD-14 5B QTL was consistent for races T2, T9, and T39, suggesting a single major genetic factor with broad resistance. The present results indicated that *QUt.mrc-5B* and *Ut6* were different genes since they were approximately 100 cM apart on Diamont/TD-14 linkage group 5B.1. The majority of resistance studies carried out thus far have also indicated a simple genetic basis for loose smut resistance, with resistance being governed by major genes [15, 16].

TD-14 carries the race-specific resistance gene *Ut11*. This gene mapped to the distal end of chromosome arm 7BS and conditioned resistance to *U. tritici* race T2, but not races T9 and T39. Knox et al. [14] attributed QTL on chromosome 7B to the major gene *Ut4*. Resistance at this locus was identified in the cultivar Glenlea (*QUt.spa-7B*, Glenlea/Taber population, at 50.4 cM, resistance to races T2, T9, and T15), in the line TD12A (*QUt.spa-7B*, Diamont/TD12A population, at 39.6 cM, resistance to races T2 and T9), and in the line 9340-CP* (*QUt.spa-7B*, 9340-CP*/AC Vista population, at 35.9 cM, resistance to race T9). *Ut11* and *Ut4* are approximately 82 cM apart on the Diamont/TD-14 chromosome 7B map based upon BLAST locations of *Ut4*-linked markers and the 90 K Infinium SNPs in the IWGSC Chinese Spring reference genome RefSeq v1.0 [26]. These results indicated that *Ut11* and *Ut4* are different genes. In addition, race T9 is avirulent on *Ut4* and virulent on *Ut11*.

Knox et al. [14] suggested that no single major gene from highly resistant cultivar Glenlea conferred resistance to all races found on the prairies of western Canada. Similarly, the broad loose smut resistance in the differential wheat line TD-14 (i.e. Sonop) is caused by multiple resistance loci. Identification of *QUt.mrc-5B* and *Ut11* in TD-14 indicated that two major genes in TD-14 conferred resistance to race T2, whereas one major gene (*QUt.mrc-5B*) was effective against races T9 and T39.

Partial infection of the heads (Fig. 1) was a significant feature of smut symptoms to races T2, T9, and T39 in the Diamont/TD-14 population. As reported previously [24], partial infection was characterized by smutting of the basal parts of spikes with the distal parts producing normal grains. The only QTL for partial smut resistance (*QUtp.mrc-5B*) was on chromosome 5B and it coincided with *QUt.mrc-5B,* which was identified based on loose smut incidence (LSI). Therefore, the same locus affected both incidence and partial smutting of heads. The PVE was higher for PLSR than for LSI when inoculated with race T2. Little has been published on the genetics or mechanisms of partial loose smut resistance in wheat. Do Valle Ribeiro [29] suggested that partial symtoms were caused by a differential growth rate between the developing heads and mycelia. The ability of the 5B QTL to impart PLSR and reduced LSI is interesting because it allows the partial resistance phenotype to be a selective marker for the 5B loose smut resistance QTL in wheat breeding programs.

## Conclusions

This study described the genetic basis of loose smut resistance in the cultivar Sonop, which is the loose smut differential line TD-14. The complete resistance phenotype of TD-14 to loose smut race T2 and strong resistance to races T9 and T39 indicated that TD-14 possesses a very effective form of resistance. We also identified and designated *Ut11* as a major loose smut resistance gene in TD-14. *Ut11* confers resistance to *U. tritici* race T2 but not races T9 and T39. *QUt.mrc-5B* provided resistance to all three races, although it was less effective against race T2, and was responsible for the partially smutted phenotype. The genes and SNP markers associated with *QUt.mrc-5B* and *Ut11* QTL have potential for use in marker-assisted selection in spring wheat breeding programs.

## Methods

### Plant material and phenotyping

A doubled haploid (DH) population of 163 lines derived from the cross between wheat lines Diamont and TD-14 was used to study the genetics of resistance to *U. tritici*. TD-14 (Sonop, pedigree = Kleintrou/Pelgrim) is a South African spring wheat variety that carries a high level of resistance to loose smut and is one of the accessions in the Canadian loose smut differential set [8], whereas Diamont (pedigree = Yubilei/Sadovo 1, synonymous with Diamant 1) is a loose smut differential line (D-6) from the former Soviet Union and susceptible to most races of the loose smut pathogen [30]. TD-14 and Diamont have been used as check lines for loose smut pathology studies for over 30 years and seed of these lines is available upon request from Morden Research and Development Centre, Morden, Canada.

The wheat parents and progenies were inoculated with *U. tritici* races T2, T9 and T39 individually in growth cabinets at Morden and Swift Current, Canada, and loose smut incidence (%) was assessed [7, 9]. Races T2, T9, and T39 were selected to reflect pathogen virulence from the Canadian prairies. Florets of the parents and mapping population DH lines were inoculated with a teliospore suspension of individual *U. tritici* races at mid-anthesis. Independently at each location, two or three spikes were inoculated per isolate on each wheat line and each spike was tagged to record the race inoculated on the spike. Growth cabinet conditions were a 21 °C day and 17 °C night, with a 16 h photoperiod. Care was taken to ensure races were kept pure, by preparing inoculum suspensions separately and keeping each race and syringe in its own sealed container. Only one race was inoculated at a time and spikes were kept separated. Inoculated spikes were harvested at maturity and the seeds from the inoculated spikes of the same race and line were combined in envelopes labelled with the race and wheat line identity.

A minimum of 30 inoculated seeds for each DH line x race combination were planted in 1 m rows in soil beds in the greenhouse such that most plants produced a single spike. Occasionally a plant had one fully smutted spike and one healthy spike, and were considered fully smutted in such cases. The greenhouse conditions for the growth of plants used in the evaluations of loose smut symptoms were a 16 h photoperiod, with supplemental lighting during the light period maintained at least 30 cm above the top of the plants. The temperature in the greenhouse ranged from 18 to 25 °C, and plants were provided with 200 mg l^− 1^ of 20–20-20 (N-P-K) at the time of watering. Loose smut incidence (LSI) in each line for each race was determined as follows:

$$ \mathrm{LSI}\ \left(\%\right)=\frac{\mathrm{Number}\ \mathrm{of}\ \mathrm{smutted}\ \mathrm{plants}}{\mathrm{Total}\ \mathrm{number}\ \mathrm{of}\ \mathrm{plants}}\times 100 $$

Partial loose smut resistance (PLSR) to *U. tritici* races T2, T9 and T39 was also studied. The number of partially and fully smutted plants of the DH population were counted (Fig. 1). When mapping PLSR, DH lines with low loose smut incidence of 0–20% were excluded since the partial smutting phenotype could not be accurately quantified in DH lines with few smutted spikes. DH lines with 20–100% incidence were included in this analysis and Fully Smutted Index (FSI) of those lines was calculated as follows:

$$ \mathrm{FSI}\ \left(\%\right)=\frac{\mathrm{Number}\ \mathrm{of}\ \mathrm{fully}\ \mathrm{smutted}\ \mathrm{plants}}{\mathrm{Total}\ \mathrm{number}\ \mathrm{of}\ \mathrm{smutted}\ \mathrm{plants}}\mathrm{x}\ 100 $$

### Genotyping

The DNeasy 96 Plant Kit (Qiagen, Toronto, Canada) was used to extract genomic DNA from lyophilized leaf tissue, which was quantified using the PicoGreen stain method (Molecular Probes, Inc., Eugene, Oregon, USA). SNP marker genotyping was performed on the parents and DH lines using the 90 K wheat Infinium SNP assay (Illumina, San Diego, CA) [31, 32]. The raw data were analyzed with the genotyping module of GenomeStudio V2011.1 software (Illumina, San Diego, USA) using default clustering parameters.

### Construction of genetic map

SNPs were filtered to exclude those with poor quality data before constructing genetic maps. SNPs with > 10% missing data were excluded from linkage analysis. Each marker was tested for deviation from the expected 1:1 ratio using chi-squared analysis. Markers showing significant (*p* < 0.01) segregation distortion were discarded. Markers were placed into preliminary linkage bins using the BIN module in QTL IciMapping version 4.1.0.0 [27, 28]. A single marker was selected from each linkage bin with the least missing data and linkage mapping was performed with MapDisto version 1.7.7 [33]. A minimum LOD score of 4.0 and a maximum recombination fraction of 0.2 was used to identify linkage groups. Loci were ordered using a combination of the “AutoMap,” “Order sequence,” and “Compare all order” functions. The best order of markers was generated using both “Auto Check Inversions” and “Auto Ripple” commands. The “Branch and Bound II” and “Seriation II” ordering methods were used in combination with the sum of adjacent recombination fractions (SARF) and count of crossover events (COUNT) as fitting criteria. For each linkage group, the shortest linkage map was selected from the linkage map solutions generated using these different mapping algorithms and criteria. Recombination fractions were converted to map distances using the Kosambi mapping function [34]. Linkage groups were assigned to chromosomes based on existing high-density SNP maps of wheat [31, 35]. DNA markers and candidate genes were located on IWGSC Chinese Spring RefSeq v1.0 [26] by BLAST. The candidate genes included the major loose smut reistance genes, *Ut4* on chromosome 7B [14, 21] and *Ut6* on chromosome 5B [14, 23].

### QTL analysis

QTL analysis was conducted with interval mapping (IM) and inclusive composite interval mapping (ICIM) using QTL IciMapping version 4.1.0.0 [27, 28]. QTL analyses were carried out for each *U. tritici* race using LSI data, as well as for the mean LSI data. The LSI data from Morden and Swift Current were combined to obtain mean LSI data for QTL analysis after plotting the data and determining that the distributions were consistent. Frequency distributions of LSI and FSI for each race in the Diamont/TD-14 DH population were plotted. Main effect QTL statistics were calculated every 0.1 cM. Permutation analysis (1000 permutations per trait) was conducted to determine the LOD significance threshold (*P* < 5%) for declaring QTL. This IM and ICIM threshold was LOD 3.2. QTL were declared when the IM or ICIM LOD statistic was greater than the LOD significance threshold in the two individual experiments (i.e. Morden and Swift Current), or the pooled dataset and at least one experiments. For these significant QTL, QTL statistics were also reported for experiments in which the LOD score was greater than 2.5. The proportion of phenotypic variance explained by single QTL was determined by the square of the partial correlation coefficient (R^2^). Analysis for epistatic QTL was conducted with 2.0 cM steps. Epistatic QTL were declared when the IM or ICIM LOD score was greater than 3.5 in the two individual experiments plus the pooled dataset.

## Supplementary information

**Additional file 1: Table S1.** Full linkage map for the Diamont/TD-14 DH population, including chromosome assignment and map position of 11,519 loci; **Table S2.** Distribution of loci in the linkage map of the Diamont/TD-14 DH population; **Table S3.** Di-genic epistatic QTL analysis for the Diamont/TD-14 DH population; **Table S4.** Linkage map and phenotypic data used for QTL analyses.

## Data Availability

The linkage map, DNA marker scoring data, and phenotypic data for the DH population is reported in Table S4. A reference genetic stock (DH line TD14XDIA*B0075) for the newly identified loose smut resistance gene *Ut11* is archived at Plant Gene Resources of Canada (PGRC) genebank as accession CN 120264. Seed of TD-14 and Diamont is available from the authors upon request.

## Abbreviations

BLAST: Basic local alignment search tool
DH: Doubled haploid
FSI: Fully smutted index
ICIM: Inclusive composite interval mapping
IM: Interval mapping
LOD: Logarithm of odds
LSI: Loose smut incidence
PLSR: Partial loose smut resistance
PVE: Phenotypic variation explained
QTL: Quantitative trait loci
SNP: Single nucleotide polymorphism
